# Models Used in Clinical Decision Support Systems Supporting Healthcare Professionals Treating Chronic Wounds: Systematic Literature Review

**DOI:** 10.2196/diabetes.8316

**Published:** 2018-06-21

**Authors:** Clara Schaarup, Louise Bilenberg Pape-Haugaard, Ole Kristian Hejlesen

**Affiliations:** 1 Department of Health Science and Technology, Aalborg University Aalborg East Denmark

**Keywords:** clinical decision support systems, statistical model, neural networks, logistic models, linear models, foot ulcer, diabetes, health personnel, systematic review, chronic wounds

## Abstract

**Background:**

Chronic wounds such as diabetic foot ulcers, venous leg ulcers, and pressure ulcers are a massive burden to health care facilities. Many randomized controlled trials on different wound care elements have been conducted and published in the Cochrane Library, all of which have only a low evidential basis. Thus, health care professionals are forced to rely on their own experience when making decisions regarding wound care. To progress from experience-based practice to evidence-based wound care practice, clinical decision support systems (CDSS) that help health care providers with decision-making in a clinical workflow have been developed. These systems have proven useful in many areas of the health care sector, partly because they have increased the quality of care, and partially because they have generated a solid basis for evidence-based practice. However, no systematic reviews focus on CDSS within the field of wound care to chronic wounds.

**Objective:**

The aims of this systematic literature review are (1) to identify models used in CDSS that support health care professionals treating chronic wounds, and (2) to classify each clinical decision support model according to selected variables and to create an overview.

**Methods:**

A systematic review was conducted using 6 databases. This systematic literature review follows the Preferred Reporting Items for Systematic Reviews and Meta-Analyses statement for systematic reviews. The search strategy consisted of three facets, respectively: Facet 1 (Algorithm), Facet 2 (Wound care) and Facet 3 (Clinical decision support system). Studies based on acute wounds or trauma were excluded. Similarly, studies that presented guidelines, protocols and instructions were excluded, since they do not require progression along an active chain of reasoning from the clinicians, just their focus. Finally, studies were excluded if they had not undergone a peer review process. The following aspects were extracted from each article: authors, year, country, the sample size of data and variables describing the type of clinical decision support models. The decision support models were classified in 2 ways: quantitative decision support models, and qualitative decision support models.

**Results:**

The final number of studies included in the systematic literature review was 10. These clinical decision support models included 4/10 (40%) quantitative decision support models and 6/10 (60%) qualitative decision support models. The earliest article was published in 2007, and the most recent was from 2015.

**Conclusions:**

The clinical decision support models were targeted at a variety of different types of chronic wounds. The degree of accessibility of the inference engines varied. Quantitative models served as the engine and were invisible to the health care professionals, while qualitative models required interaction with the user.

## Introduction

### Background

Chronic wounds such as diabetic foot ulcers, venous leg ulcers, and pressure ulcers are a massive burden on the health care facility [1-4]. The costs of treating chronic wounds are considerable, among other things because of the length and complexity of ulceration [2,5,6]. In Denmark, wound care of chronic wounds is organized by specialized hospital units, general practitioners, nursing clinics in municipalities and community nurses visiting people with chronic wounds in their homes [7].

Conventional care of chronic wounds includes [1,2,4,8-11]: debridement, off-loading, antibiotic treatment in case of infection and add-on therapies such as negative-pressure wound therapy. Many randomized controlled trials on these elements have been conducted and published in the Cochrane Library [12-20]. The conclusion of almost all of the randomized controlled trials is that health care professionals are forced to rely on their own experience when making decisions regarding the treatments. A recent study [21] from 2017 has likewise examined community nurses’ professional basis for treating diabetic foot ulcers and found that they have to rely on experience and to ask colleagues for advice when undertaking wound care.

In an attempt to increase the evidential basis and help nurses proceed from experience-based practice to evidence-based practice, several alternatives have been considered. One of these involves the development and application of health information technology systems [22,23]. An example of a health information technology system is clinical decision support systems (CDSS), which are characterized by their ability to analyze data to enhance health care providers’ ability to make decisions in a clinical workflow [24,25]. According to van Bemmel and Musen [26] the models used in CDSS are either quantitative decision support models or qualitative decision support models, depending on the techniques that are applied in the clinical decision support model. The techniques applied in quantitative decision support models are typically based on well-defined statistical processes and make use of training sets of patient data. Thus, it becomes objective and reproducible [26]. The techniques applied in qualitative decision support models frequently use features that have been proposed by experts and are based on clinical studies [26,27].

**Figure 1:**
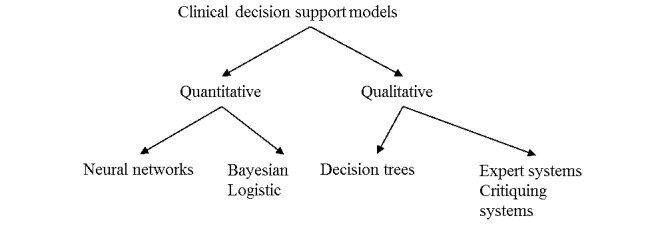
Clinical decision support models can be grouped according to different classifications. Included here are examples of the different approaches related to each classification.

As it can be seen in Figure 1, examples of the different approaches from left to right range from data-intensive approaches to knowledge-intensive approaches. According to Shortliffe et al [27] it is possible to distinguish between four types of knowledge: (1) knowledge derived from data analysis, (2) judgemental or subjective knowledge, (3) scientific or theoretical knowledge, and (4) high-level strategic knowledge or “self-knowledge”. These authors elaborate on their understanding of “judgmental” knowledge as follows: “*experience and opinions of experts regarding an issue about which the formal data may be fragmentary or non-existent* ” [27]. In this systematic literature review, expert systems will be considered in the light of Shortliffe et al [27] and their understanding of judgemental knowledge.

Over the decades, CDSS have proven to be useful in many areas within the health care sector [23-25,28-33], partly because CDSS have increased the quality of care provided, and partly because they generate a solid basis for evidence-based practice. In the Bright et al [23] systematic review from 2012, the goal was to evaluate the effect of CDSS on clinical outcomes, health care processes, workload and efficiency, patient satisfaction, cost and provider’s use and implementation. In total, Bright et al [23] systematically reviewed 148 randomized, controlled trials. They concluded that CDSS could improve health care process measures; however, the evidence regarding all the other areas they investigated was sparse. In the Blum et al [30] systematic review from 2014, the literature was systematically reviewed for content and application of computer-based CDSS, and their effects on patient-reported outcome were considered. Fifteen studies were included in this review. Blum et al [30] reported no negative effects related to patient-reported outcomes. At the same time, they described marginally positive effects of CDSS on specific patient-reported outcomes [30]. Both systematic reviews had a particular focus on clinical outcomes. None of the systematic reviews focused on CDSS within the field of diabetic foot ulcer care. In fact, it was not possible to identify any overview of existing CDSS within the area of diabetic foot ulcer wound care.

The aims of this systematic literature review are (1) to identify models used in CDSS from the past decade that support health care professionals treating chronic wounds, (2) to classify each clinical decision support model, and (3) to create an overview.

## Methods

### Protocol Registration

The present systematic literature review follows the Preferred Reporting Items for Systematic Reviews and Meta-Analyses (PRISMA) statement for systematic reviews [34]. The protocol for this systematic literature review was registered on the online prospective review database (PROSPERO) with the registration no.: CRD42017068495 [35]. We used the PRISMA checklist, which is an author guidance for reporting systematic reviews to report the 10 studies. We could have used the Consolidated Standards of Reporting Trials CONSORT checklist, however, that specific author guidance is more applicable for reporting randomized trials.

### Information Sources

Publications from MEDLINE/PubMed, Cumulative Index of Nursing and Allied Health Literature (CINAHL), The Cochrane Library, Excerpta Medica dataBASE (EMBASE), Scopus and Web of Science were searched in March 2017 to identify articles that described and discussed clinical decision support models supporting health care professionals treating chronic wounds.

### Search Strategy

The selected databases used different terminology in indexing articles, meaning that there is a risk that not all relevant articles are identified during a search. In an attempt to include all relevant articles, we used thesauruses, a systematic record in databases of subject headings used to index articles. To organize the search systematically, we grouped the search terms around three facets: ‘algorithm’, ‘wound care’, and ‘clinical decision support systems’. Further elaboration of the search terms used for eligible articles in the three facets can be seen in Table 1. The search strategy consisted of three facets, respectively: Facet 1 (Algorithm), Facet 2 (Wound care) and Facet 3 (Clinical decision support system). The terms within each facet were a mix of Medical Subject Headings (MeSH) terms and synonyms. Between each facet, the Boolean operator AND was applied, and between each MeSH term and synonyms the Boolean operator OR was applied. Only a few limitations were marked in the search criteria. Limitations included studies written in languages other than English, literature published before 2006 and studies conducted on animals and children. In the databases, children were defined as subjects younger than 19 years old. We were not interested in wound care algorithms related to animals since wound healing in animals may differ from wound healing processes in human beings.

### Inclusion and Exclusion Criteria

The focus of this study was on models used in CDSS within the area of wound care related to chronic wounds. We, the author and the co-authors, were interested in studies that presented algorithms, models, and that were relevant for wound care as well as studies that presented wound care decision support systems or clinical decision support models. Since wound care differs depending on whether the wound is acute or chronic, we excluded studies based on acute wounds or trauma.

We excluded studies that presented guidelines, protocols, and instructions focusing on wound care since they do not require progression along an active chain of reasoning from the clinicians, just their focus. Furthermore, clinical decision support models serve as learning tools, which was of interest to us. Finally, studies were excluded if they had not undergone a peer review process.

### Study Selection and Data Extraction

The reference management software program Legacy Refworks (version 2.0, 2010) was used to handle the articles. In order to remove duplicates in the identified references, the functions ‘Exact Duplicates’ and ‘Close Duplicates’ were applied.

Titles and abstracts of the identified citations were read to screen the articles using the inclusion and exclusion criteria described in the previous section. The articles that remained were then read in full to extract relevant information. Afterward, cross-referencing techniques were applied on the reference lists of the included articles to identify literature that had not been discovered through the systematic literature search. The extracted information included authors, year, country, a sample size of the data, and variables describing the type of clinical decision support model [26].

**Table 1:** The three facets below, shows the search strategy applied in the systematic literature review. Each facet consists of MeSH terms and synonyms. Between each MeSH term and synonym, the Boolean operator OR is used and between each facet the Boolean operator AND is applied.

Facet 1 (Algorithm)		Facet 2 (Wound care)		Facet 3 (Clinical decision support system)
Regression analysis OR Statistical models OR Linear models OR Loglinear model OR Multivariate logistic regression OR Logistic models OR Regression analysis OR Logistic regression OR Artificial neural network OR Theoretical model OR Computer simulation OR Prediction OR Bayes theorem OR Prognosis OR Forecasting OR Artificial intelligence OR Artificial intelligence OR Algorithm-based OR Model-based OR Model OR Algorithms OR Prescriptive OR Pattern recognition OR Data mapping OR Text mining OR Data mining	AND	Therapy OR Wound treatment OR Wound management OR Wound assessment OR Pressure ulcer care OR Wound care OR Skin care OR Skin care OR Foot care OR Larval therapy OR Autolytic debridement OR Chemical debridement OR Mechanical debridement OR Surgical debridement OR Debridement	AND	Clinical decision support systems

To reduce bias during the selection and reviewing process, the author, together with one of the co-authors, systematically went through each article, discussed the scope of each article and decided whether an article was relevant in proportion to our systematic literature review. The interrater reliability was not calculated in this study. However, it could have been prudent.

The included models used in the CDSS from the studies were subsequently described and classified according to selected variables, as defined by van Bemmel and Musen [26] and supplemented with components from Shortliffe et al [27]. The models were classified in two ways: (1) quantitative decision support models, and (2) qualitative decision support models (Figure 1).

## Results

### Study Selection

**Figure 2:**
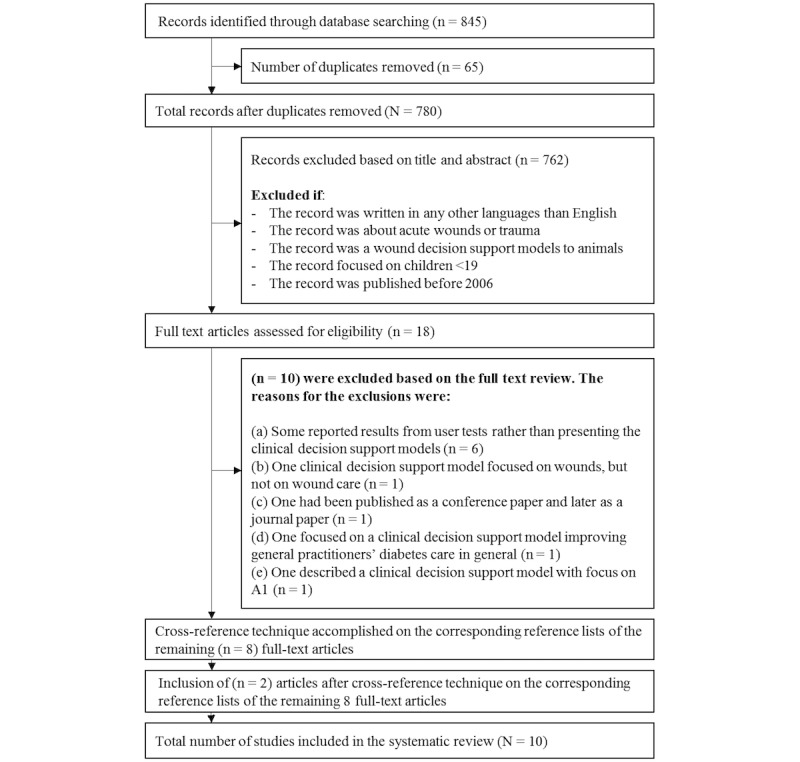
The flowchart visualises the selection process of the articles included in the systematic literature review.

Figure 2 depicts the flowchart of the selection process of articles included in the systematic literature review. Systematic searches led to the identification of 845 articles. Before starting the preliminary screening process of titles and abstracts, we removed 65 duplicates, ending up with 780 records to screen. The screening process followed the inclusion and exclusion criteria, as explained in the method section, leaving 18 articles for full-text review. There were 10 articles excluded based on the full-text review process. The corresponding reference lists of the remaining 8 full-text articles were reviewed in the same way as the full-text articles had been. This extra step resulted in the identification of 2 additional articles. Hence, the final number of studies included in the systematic review was 10. The earliest relevant article was published in 2007, and the most recent was from 2015.

### Quantitative Decision Support Models

A total of 4 of the 10 (40%) articles from the systematic literature review presented a quantitative decision support model [36-39]. The oldest of the 4 studies was published in 2010, and the newest was from 2015. Of these, 2 studies were published in Spain, and the remaining 2 were published in India and the United States (Table 2). All 4 studies present quantitative decision support models as proof of concepts.

The applied techniques in these 4 quantitative decision support models covered the following: Neural Networks, Support Vector Machines, Random Forest Decision Tree, Bayesian Belief Networks and Logistic Regression (Table 3). The data sets applied in the clinical decision support models encompassed the sizes (n=113, n=73, n=74, n=113) and 3 of the 4 (75%) data sets involved images of wounds [36,38,39]. Only in 1 of the 4 (25%) quantitative decision support models did the data set comprise demographic and clinical information such as gender, age, body mass index, tobacco use, instead of wound images [37].

The professionals who had access to 3 of the 4 (75%) quantitative decision support models were health care professionals who detect, estimate, diagnose, and register crucial tissue measurements for pressure ulcer diagnosis. They are also health care professionals who undertake wound care for chronic wounds (Table 4), [36,38,39]. The last quantitative decision support model by Forsberg et al [37] was applicable only for surgeons working in a hospital setting who needed to improve their medical decision-making.

The accessibility of the inference engines of the 4 quantitative decision support models was low. The 4 quantitative decision support models should help health care professionals in decision-making situations, but health care professionals could not follow the statistical processes performed on the data material by personal inspection. They could only see the outcome of the statistical processes (Table 4).

The focus in each of the 4 quantitative decision support models varied. The quantitative decision support model by Veredas et al [36] from 2015 presented a clustering-based image segmentation approach along with statistical methods to accomplish automatic tissue recognition for pressure ulcer diagnosis. The decision support model helped health care professionals in making decisions, but was only the engine and therefore was not available or visible to the health care professionals. The quantitative decision support model by Forsberg et al [37] from 2015 combined biomarker data with clinical observations and generated predictive algorithms that helped surgeons identify when to close or otherwise cover wounds in high-risk military and civilian populations. Similar to the previous decision support model by Veredas et al [36] from 2015, the model by Forsberg et al [37] was also part of the engine and hence not visible to health care professionals.

**Table 2:** An overview of who the publication authors were, the year the publication was published and where the publication was published.

Reference No.	Publication Authors	Year Published	Country Where Published
[36]	Veredas FJ, Luque-Baena RM, Martín-Santos FJ, Morilla-Herrera JC, Morente L	2015	Spain
[37]	Forsberg JA, Potter BK, Wagner MB, Vickers A, Dente CJ, Kirk AD, Elster EA	2015	US
[38]	Mukhejerjee R, Manohar DD, Das DK, Achar A, Mitra A, Chakraborty C	2014	India
[39]	Veredas F, Mesa H, Morente L	2010	Spain

**Table 3:** The table provides an overview of which type and size of data the models were based on, and the applied techniques in the clinical decision support systems.

Reference No.	Data Presented in the Article	Applied techniques in the clinical decision support systems
[36]	Data consisted of (n=113) images of pressure ulcers on sacrum and hips.	K-means clustering algorithm for image segmentation. Three machine learning approaches (1) Neural Networks, (2) Support Vector Machines, and (3) Random Forest Decision Trees
[37]	Data consisted of (n=73) participants (a mix of soldiers and civilians) with at least one extremity wound >75cm^2^.	Parametric statistical and machine learning methodologies (1) Bayesian Belief Networks, (2) Random Forest Analysis, and (3) Logistic regression using Least Absolute Shrinkage and Selection Operator. Statistical differences between the continuous variables and wound outcomes were evaluated using the Mann-Whitney U test and the post hoc Tukey-Kramer assessment.
[38]	Data consisted of (n=74) images of chronic wounds from the Medetec medical image database.	Fuzzy divergence-based thresholds used for wound contour segmentation. For wound tissue classification (1) Bayesian classification, and (2) Support vector machine.
[39]	Data consisted of (n=113) images of sacrum and hip pressure ulcers.	Image processing techniques: filtering, kernel smoothing by the mean shift procedure and region growing. Statistical analysis: (1) A hybrid approach based on Neural networks, and (2) Bayesian classifiers.

**Table 4:** An overview of the quantitative decision support models’ accessibility of the inference engines, what type of wounds it focuses on and the type of professionals who have access.

Reference No.	Accessibility of the inference engines of the system	Type of Wounds	Professionals, who have access to the system
[36]	The clinical decision support model aims to help clinicians in decision-making situations. Health care professionals cannot access the inference engine and cannot follow the statistical processes performed on the data by personal inspection. They can only see the outcomes of the statistical processes.	Pressure ulcers	Health care professionals who detect, estimate, diagnose and register important tissue measurements for pressure ulcer diagnosis
[37]	The clinical decision support model aims to improve decision-making when surgeons need to know if they must close or cover a wound. Surgeons cannot access the inference engine and cannot follow the ongoing statistical processes in the decision support model. They can only see the outcomes of the statistical processes.	Chronic wounds	Surgeons in hospital settings
[38]	The decision support model helps health care professionals identify necrotic tissue within chronic wounds. Clinicians cannot access the inference engine. They can only see the outcomes of the statistical processes.	Chronic wounds	Health care professionals who undertake wound care for chronic wounds
[39]	The decision support model helps health care professionals care for pressure ulcers. The health care professionals cannot access the inference engine and cannot follow statistical processes. They can only see the outcomes of the statistical processes.	Pressure ulcers	Health care professionals who detect, estimate, diagnose and register important tissue measurements for pressure ulcer diagnosis

The quantitative decision support model by Mukherjee et al [38] from 2014 was a clinical decision support model that could identify necrotic tissue in chronic wounds. Like the 2 previous decision support models, Mukherjee’s model helped health care professionals, but the statistical processes were again not visible to health care professionals, as only the outcomes of the statistical processes were shown. The last quantitative decision support model by Veredas et al [39] from 2010 was also an engine that could recognize tissue in pressure ulcer images, and it was therefore also invisible to health care professionals.

Pressure ulcers were the focus of 2 of the studies [36,39]; the remaining 2/4 (50%) focused on chronic wounds [37,38].

### Qualitative Decision Support Models

There were 6 articles (6/10, 60%)from the systematic literature review that presented a qualitative decision support model [40-45]. The oldest article was published in 2007, and the newest was published in 2015. Five of the six studies (83%) were published in the United States while the remaining study (1/6, 17%) was published in Great Britain (Table 5). Five of the six studies (83%) present qualitative decision support models as proof of concepts. Only the study by Smith and Gibson [42] from 2013 present a qualitative decision support model which the health care professionals used.

The applied techniques in the 6 qualitative decision support models included Meta-Analyses, Systematic Reviews, Literature Reviews, Expert Face Validations, Answers from Questionnaires, Expert Panel Discussions, Task Force of Clinical Experts and Consensus Panels (Multimedia Appendix 1). The techniques covered among other things that experts are in charge of proposing features when building qualitative decision support models, and models are based on clinical studies with the highest evidence [26]. In 3 of the 6 (50%) qualitative decision support models, the techniques were a combination of literature reviews and expert panel discussions [41,44,45]. Two of the remaining 3 (67%) qualitative decision support models used only one of the mentioned techniques [42,43], and the applied technique in the last qualitative decision support model (1/3, 33%) was not described in the study [40].

The professionals who had access to 3 of the 6 (50%) qualitative decision support models were health care professionals who undertake wound care for chronic wounds (Multimedia Appendix 2) [41,44,45]. Two of the 6 (33%) qualitative decision support models were designed specifically for registered nurses, licensed practical nurses and specialized nurses who provide critical support for tissue viability services [40,42]. The last qualitative decision support model by Kravitz et al [43] from 2007 was applicable only for surgeons undertaking diabetic foot ulcer surgery (Multimedia Appendix 2).

**Table 5:** An overview of who the publication authors were, the year the publication was published and where the publication was published.

Reference No.	Publication Authors	Year Published	Country Where Published
[40]	Alvey B, Hennen N, Heard H	2012	US
[41]	Beitz JM, van Rijswijk L	2012	US
[42]	Smith G, Gibson E	2013	Great Britain
[43]	Kravitz SR, McGuire JB, Sharma S	2007	US
[44]	LeBlanc K, Baranoski S, Christensen D, Langemo D, Sammon MA, Edwards K, Holloway S, Gloeckner M, Williams A, Sibbald RG, Regan M	2013	US
[45]	McNichol L, Watts C, Mackey D, Beitz JM, Gray M	2015	US

The accessibility of the inference engines of the 6 qualitative decision support models was high. Health care professionals could follow the decision process from start to finish; furthermore, they were required to take an active part in assessing the wounds.

The focus of each of the 6 qualitative decision support models varied. In the qualitative decision support model of Alvey et al, [40] registered nurses and licensed practical nurses were required to select descriptive information on a wound to let the qualitative decision support model assist them during decision-making. The qualitative decision support models by Beitz and Rijswijk [41] were relevant in three different areas: initially assessing a wound, increasing the wound assessment at every dressing change of surgical and acute wounds, and improving wound assessment for chronic wounds at every dressing change. The qualitative decision support model of Smith and Gibson [42] required the wound care link nurses to decide on characteristics of the wound and thereby receive assistance in decision making. The qualitative decision support model by Kravitz et al [43] required surgeons to choose between proactive (elective) diabetic foot ulcer surgery and reactive (nonelective) diabetic foot ulcer surgery. The qualitative decision support model by LeBlanc et al [44] was designed to be used in multiple health care settings and by all levels of staff and caregivers. The last qualitative decision support model by McNichol et al [45] was also designed to be used by multiple health care professionals, and clinicians could interact with three different models: a skin and pressure ulcer risk assessment algorithm, a prevention of pressure ulcers algorithm, and a treatment of pressure ulcers algorithm.

Diabetic foot ulcer surgery was the focus of 1/6 (17%) studies [43], 4/6 (67%) focused on chronic wounds [41,42,44,45], and the last 1/6 (17%) study focused on pressure ulcers [40].

## Discussion

### Principal Results

Recent studies have shown that it can be valuable to apply CDSS in clinical settings to increase the quality of care and generate solid bases for evidence-based practice [23,28-31]. Hence, the aims of this systematic literature review were to identify the various models used in CDSS over the past decade that support health care professionals treating chronic wounds, to classify each clinical decision support model and to create an overview. A total of 10 clinical decision support models were identified, 4/10 (40%) of which were quantitative and 6/10 (60%) of which were qualitative [36-45]. The clinical decision support models were targeted at different types of chronic wounds. The degree of accessibility of the inference engines varied. Quantitative decision support models served as the engine and were invisible to the health care professionals. The qualitative decision support models required health care professionals’ involvement.

### Comparison With Prior Work

Chronic wounds are complicated to treat and challenging for health care professionals. In decision-making situations where health care professionals need advice related to chronic wound treatments, health care professionals are, according to the literature, often forced to rely on their own experience rather than having evidence available that they can follow [21,46]. A cross-sectional survey by Stolt et al [47] measured nurses’ knowledge of foot care and concluded that nurses need more knowledge and have clinical knowledge gaps. Several other studies have pointed out that health care professionals’ curriculum is lacking when it comes to wound care [21,48-51]. On the basis of the studies included in the present systematic literature review, one might say that the conditions the health care professionals have are not as good as they could be when it comes to performing evidence-based practice [36-45]. The deficient number of studies could indicate that there is a need to develop more clinical decision support models targeted at chronic wounds to increase the quality of care and to support evidence-based wound care practice [21,46,47].

The optimal type of decision support model may not be easy to identify. The techniques applied in the clinical decision support models reviewed here were classified into two groups: 4 studies were based on quantitative models, and 6 studies were based on qualitative models. One of the strengths of the quantitative decision support model classification is that outcomes of statistical processes are objective, reproducible and in accordance with the clinical data [26]. However, applying the models can be somewhat complicated. The qualitative decision support models have different strengths and limitations [26]. One of the strengths of the qualitative approach is that many years of professional experience within a specific domain help experts manage unknown and uncertain situations where they use their clinical knowledge and experience and thereby build up significant judgemental and tacit knowledge [27]. When experts participate in expert panel discussions or consensus panels, it must be assumed that the basis on which they speak is well-founded. However, although experts may have many years of professional experience, there may still be gaps in their knowledge and experience. As such, 6 included qualitative decision support models may lack validity. With data being increasingly available [32,52-54], the argument for using a quantitative approach is strengthened, and one might suggest that future work focuses to a greater extent on quantitative techniques.

It is commonly accepted that accessibility of the inference engine is important [55]. Quantitative and qualitative decision support models differ from each other in this respect. The quantitative decision support models appeared as engines without requiring involvement, whereas the qualitative decision support models required health care professionals to take an active part and be involved. In other words, systems based on qualitative models are more accessible than systems based on quantitative models. Although there are fewer accessible models in quantitative systems, the advantage of using Big Data on large data sets may outweigh the disadvantages related to accessibility [52,53]. Future research should investigate how to compensate for lack of accessibility in quantitative systems by developing methods for augmenting users’ understanding of the decision processes.

One might argue that IT systems should be tailored to specific professional groups to address the needs of nurses or doctors optimally, for example [31]. Some systems found in our review focused solely on supporting surgeons, some supported wound specialists and some focused on no specific profession. The heterogeneity of the target group of professionals may be explained by the fact that treating chronic wounds is a multidisciplinary task, requiring the participation of many different professions [16]. Future research should include work on how to balance the two concerns—facilitating the need for multidisciplinary collaboration as well as optimally addressing the specific needs of each professional group.

### Limitations

Several of the existing checklists are addressed to conventional study designs such as randomized controlled trials, cohort studies and qualitative semi-structured interviews [56]. However, none of the scientific articles identified in our systematic literature review applied any of the mentioned study designs.

It is a complicated task to build a search strategy which reflects an accurate inventory of what has been done within a research area. Primarily because hits often reflect the conducted search rather than reflecting the reality within a research area. However, there are several initiatives to avoid this problem. In our study, our preliminary search strategy consisted of 4 facets, respectively: Facet 1 (Algorithm), Facet 2 (Wound care), Facet 3 (Clinical decision support system) and Facet 4 (Wound). Facet4 consisted of a mix of MeSH terms and synonyms and these were: “foot ulcer,” “diabetic foot,” “skin ulcer,” “leg ulcer,” “decubitus,” “chronic wound,” “venous foot,” “venous ulcer,” “pressure ulcer,” “ulcer,” “wounds and injuries,” “varicose ulcer,” “ulcer wound,” and “diabetic foot”. Between each of the search terms in Facet 4 the Boolean operator “OR” was inserted to achieve as many hits as possible. When running the search with the 4 facets, we did not receive any hits, maybe because it was too narrow. Hence, we refined our search strategy and made it broader. Subsequently, we removed search query facet 4, so the search strategy instead only consisted of Facet1, Facet2 and Facet3. When running the revised search strategy, we retrieved several hits as depicted in our flowchart (Figure 2). We could have refined our search strategy further, so it only consisted of Facet2 and Facet3, and thereby we would have received more than 9600 hits. One might think that the risk of missing and identifying potential articles is rather high when more than 9600 articles should be read through by the human eye.

We used the classification suggested by Bemmel and Musen [26] of clinical decision support models discriminating between quantitative models and qualitative models. However, other classifications might have been relevant as well.

### Conclusions

There were 10 clinical decision support models identified. Of these, 4 (40%) were quantitative decision models and 6 (60%) were qualitative decision support models.

Three (3/4, 75%) of the quantitative decision support models were applicable for all health care professionals who detect, estimate, diagnose and register essential tissue measurements for pressure ulcer diagnosis or who undertake wound care for chronic wounds. The fourth (1/4, 25%) quantitative decision support model was applicable for surgeons who work in a hospital setting. Two qualitative decision support models were designed specifically for registered nurses, licensed practical nurses and specialized nurses such as wound care nurses who provide critical support for tissue viability services. One qualitative decision support model applicable for surgeons who undertake diabetic foot ulcer surgery.

The degree of accessibility of the inference engines varied. The 4 quantitative decision support models served as engines and were invisible to health care professionals. The 6 qualitative decision support models required interaction with health care professionals.

The clinical decision support models were targeted towards different types of chronic wounds. Two (2/4, 50%) of the quantitative decision support models focused on pressure ulcers, while the remaining 2/4 (50%) focused on chronic wounds. One of the 6 (17%) qualitative decision support models explicitly focused on diabetic foot ulcer surgery, 4 (4/6, 67%) focused on chronic wounds, and the last (1/6, 17%) qualitative decision support model focused on pressure ulcers.

More research is needed to develop clinical decision support models targeted at health professionals treating chronic wounds. Given the growing focus on evidence-based care and the availability of increasing amounts of data, the arguments for a quantitative approach to decision models in future work are strengthened. Future research should also address problems with accessibility in quantitative systems by developing methods for augmenting users’ understanding of the processes in the quantitative models.

## Appendix

Multimedia Appendix 1 The table provides an overview of which type and size of data the models were based on, and the applied techniques in the clinical decision support systems.

Multimedia Appendix 2 An overview of the qualitative decision support models’ accessibility of the inference engines, what type of wounds it focuses on and the type of professionals who have access.

## Abbreviations

CDSS: Clinical Decision Support Systems
CINAHL: Cumulative Index of Nursing and Allied Health Literature
CONSORT: Consolidated Standards of Reporting Trials
EMBASE: Excerpta Medica Database
MEDLINE: Medical Literature Analysis and Retrieval System Online
MeSH: Medical Subject Headings
PRISMA: Preferred Reporting Items for Systematic Reviews and Meta-Analyses
PROSPERO: Prospective Review Database

## References

[1] Frykberg RG, Banks J (2015). Challenges in the Treatment of Chronic Wounds. Adv Wound Care (New Rochelle).

[2] Harding KG, Morris HL, Patel GK (2002). Science, medicine and the future: healing chronic wounds. BMJ.

[3] Nussbaum S, Carter M, Fofe C, DaVanzo J, Haught R, Nusgart M, Cartwritht D (2017). An Economic Evaluation of the Impact, Cost, and Medicare Policy Implications of Chronic Nonhealing Wounds. Value in Health.

[4] Järbrink Krister, Ni G, Sönnergren Henrik, Schmidtchen A, Pang C, Bajpai R, Car J (2017). The humanistic and economic burden of chronic wounds: a protocol for a systematic review. Syst Rev.

[5] Khalil H, Cullen M, Chambers H, Carroll M, Walker J (2016). Reduction in wound healing times, cost of consumables and number of visits treated through the implementation of an electronic wound care system in rural Australia. Int Wound J.

[6] Hjort A, Gottrup F (2010). Cost of wound treatment to increase significantly in Denmark over the next decade. J Wound Care.

[7] Jørgensen S F, Nygaard R, Posnett J (2013). Meeting the challenges of wound care in Danish home care. J Wound Care.

[8] Woo KY, Botros M, Kuhnke J, Evans R, Alavi A (2013). Best practices for the management of foot ulcers in people with diabetes. Adv Skin Wound Care.

[9] Falanga V (2005). Wound healing and its impairment in the diabetic foot. The Lancet.

[10] Bekara F, Vitse J, Fluieraru S, Masson R, Runz AD, Georgescu V, Bressy G, Labbé Jean Louis, Chaput B, Herlin C (2018). New techniques for wound management: A systematic review of their role in the management of chronic wounds. Arch Plast Surg.

[11] Han G, Ceilley R (2017). Chronic Wound Healing: A Review of Current Management and Treatments. Adv Ther.

[12] Dumville JC, Deshpande S, O'Meara S, Speak K (2011). Foam dressings for healing diabetic foot ulcers. Cochrane Database Syst Rev.

[13] Han G, Ceilley R (2017). Chronic Wound Healing: A Review of Current Management and Treatments. Adv Ther.

[14] Stoekenbroek RM, Santema TB, Legemate DA, Ubbink DT, van DBA, Koelemay MJW (2014). Hyperbaric oxygen for the treatment of diabetic foot ulcers: a systematic review. Eur J Vasc Endovasc Surg.

[15] Dumville JC, O'Meara S, Deshpande S, Speak K (2011). Hydrogel dressings for healing diabetic foot ulcers. Cochrane Database Syst Rev.

[16] Alexiadou K, Doupis J (2012). Management of diabetic foot ulcers. Diabetes Ther.

[17] Bergin SM, Wraight P (2006). Silver based wound dressings and topical agents for treating diabetic foot ulcers. Cochrane Database Syst Rev.

[18] Dumville JC, O'Meara S, Deshpande S, Speak K (2012). Alginate dressings for healing diabetic foot ulcers. Cochrane Database Syst Rev.

[19] Cruciani M, Lipsky BA, Mengoli C, de LF (2013). Granulocyte-colony stimulating factors as adjunctive therapy for diabetic foot infections. Cochrane Database Syst Rev.

[20] Selva OA, Solà Ivan, Barajas-Nava LA, Gianneo OD, Bonfill CX, Lipsky BA (2015). Systemic antibiotics for treating diabetic foot infections. Cochrane Database Syst Rev.

[21] Schaarup C, Pape-Haugaard L, Jensen MH, Laursen AC, Bermark S, Hejlesen OK (2017). Probing community nurses' professional basis: a situational case study in diabetic foot ulcer treatment. Br J Community Nurs.

[22] Buntin MB, Burke MF, Hoaglin MC, Blumenthal D (2011). The benefits of health information technology: a review of the recent literature shows predominantly positive results. Health Aff (Millwood).

[23] Bright TJ, Wong A, Dhurjati R, Bristow E, Bastian L, Coeytaux RR, Samsa G, Hasselblad V, Williams JW, Musty MD, Wing L, Kendrick AS, Sanders GD, Lobach D (2012). Effect of clinical decision-support systems: a systematic review. Ann Intern Med.

[24] Coiera E (2003). Guide to health informatics. London: Arnold Busck; ISBN.

[25] Sim I, Gorman P, Greenes RA, Haynes RB, Kaplan B, Lehmann H, Tang PC (2001). Clinical decision support systems for the practice of evidence-based medicine. J Am Med Inform Assoc.

[26] Bemmel JV, Musen M (2002). Handbook of Medical Informatics.

[27] Shortliffe E, Buchanan B, Feigenbaum E (1979). Knowledge engineering for medical decision making: A review of computer-based clinical decision aids. Proc. IEEE.

[28] Kawamoto K, Houlihan CA, Balas EA, Lobach DF (2005). Improving clinical practice using clinical decision support systems: a systematic review of trials to identify features critical to success. BMJ.

[29] Delaney BC, Fitzmaurice DA, Riaz A, Hobbs FD (1999). Can computerised decision support systems deliver improved quality in primary care?. Interview by Abi Berger. BMJ.

[30] Blum D, Raj SX, Oberholzer R, Riphagen II, Strasser F, Kaasa S, EURO IMPACT‚ European Intersectorial Multidisciplinary Palliative Care Research Training (2015). Computer-Based Clinical Decision Support Systems and Patient-Reported Outcomes: A Systematic Review. Patient.

[31] Garg AX, Adhikari NKJ, McDonald H, Rosas-Arellano MP, Devereaux PJ, Beyene J, Sam J, Haynes RB (2005). Effects of computerized clinical decision support systems on practitioner performance and patient outcomes: a systematic review. JAMA.

[32] Staszewska A, Zaki P, Lee J (2017). Computerized Decision Aids for Shared Decision Making in Serious Illness: Systematic Review. JMIR Med Inform.

[33] Thomas KW, Dayton CS, Peterson MW (1999). Evaluation of internet-based clinical decision support systems. J Med Internet Res.

[34] Moher D, Liberati A, Tetzlaff J, Altman DG, PRISMA Group (2009). Preferred reporting items for systematic reviews and meta-analyses: the PRISMA statement. PLoS Med.

[35] (2017). Prospero Registration.

[36] Veredas FJ, Luque-Baena RM, Martín-Santos FJ, Morilla-Herrera JC, Morente L (2015). Wound image evaluation with machine learning. Neurocomputing.

[37] Forsberg JA, Potter BK, Wagner MB, Vickers A, Dente CJ, Kirk AD, Elster EA (2015). Lessons of War: Turning Data Into Decisions. EBioMedicine.

[38] Mukherjee R, Manohar DD, Das DK, Achar A, Mitra A, Chakraborty C (2014). Automated tissue classification framework for reproducible chronic wound assessment. Biomed Res Int.

[39] Veredas F, Mesa H, Morente L (2010). Binary tissue classification on wound images with neural networks and bayesian classifiers. IEEE Trans Med Imaging.

[40] Alvey B, Hennen N, Heard H (2012). Improving accuracy of pressure ulcer staging and documentation using a computerized clinical decision support system. J Wound Ostomy Continence Nurs.

[41] Beitz JM, van RL (2012). ﻿﻿﻿﻿﻿﻿﻿Developing evidence-based algorithms for negative pressure wound therapy in adults with acute and chronic wounds: literature and expert-based face validation results. Ostomy Wound Manage.

[42] Smith G, Gibson E (2013). The development of an algorithm to support nurses choosing dressings for chronic exudate. Wounds UK.

[43] Kravitz SR, McGuire JB, Sharma S (2007). The treatment of diabetic foot ulcers: reviewing the literature and a surgical algorithm. Adv Skin Wound Care.

[44] LeBlanc K, Baranoski S, Christensen D, Langemo D, Sammon MA, Edwards K, Holloway S, Gloeckner M, Williams A, Sibbald RG, Regan M (2013). International Skin Tear Advisory Panel: a tool kit to aid in the prevention, assessment, and treatment of skin tears using a Simplified Classification System ©. Adv Skin Wound Care.

[45] McNichol L, Watts C, Mackey D, Beitz JM, Gray M (2015). Identifying the right surface for the right patient at the right time: generation and content validation of an algorithm for support surface selection. J Wound Ostomy Continence Nurs.

[46] Haram R, Ribu E, Rustøen Tone (2003). The views of district nurses on their level of knowledge about the treatment of leg and foot ulcers. J Wound Ostomy Continence Nurs.

[47] Stolt M, Suhonen R, Puukka P, Viitanen M, Voutilainen P, Leino-Kilpi H (2015). Nurses' knowledge of foot care in the context of home care: a cross-sectional correlational survey study. J Clin Nurs.

[48] Gottrup F (2012). Education in Wound Management in Europe with a Special Focus on the Danish Model. Adv Wound Care (New Rochelle).

[49] Timmons J (2006). Wound care education needs a boost. Br J Community Nurs.

[50] Declaration Professional Bachelor's Degree in Nursing.

[51] Chamanga ET (2014). Community nurses' experiences of treating patients with leg ulcers. J Clin Nurs ?34, ISSN.

[52] Kruse Clemens Scott, Goswamy Rishi, Raval Yesha, Marawi Sarah (2016). Challenges and Opportunities of Big Data in Health Care: A Systematic Review. JMIR Med Inform.

[53] Kruse CS, Goswamy R, Raval Y, Marawi S (2016). Challenges and Opportunities of Big Data in Health Care: A Systematic Review. JMIR Med Inform.

[54] Wang W, Krishnan E (2014). Big data and clinicians: a review on the state of the science. JMIR Med Inform.

[55] Nielsen J (1993). Usability Engineering.

[56] (2017). Enhancing the QUlity and Transparency OF health Research.

