# Supplementary material for: Models Used in Clinical Decision Support Systems Supporting Healthcare Professionals Treating Chronic Wounds: Systematic Literature Review
Source: JMIR Diabetes. 2018 Jun 21;3(2):e11. doi: 10.2196/diabetes.8316 (PMC6238865; doi:10.2196/diabetes.8316)
Supplement: Multimedia Appendix 1 [file diabetes_v3i2e11_app1.pdf]

Table 6 The table provides an overview of which type and size of data the models were based on, and the applied techniques in the clinical decision support systems.

Reference No.	Data Presented in the Article	Applied techniques in the clinical decision support systems
[40]	Data consisted of (*n*=31) participants: nurses, student nurses and volunteers.	Not described in the study.
[41]	Data consisted of (N=34) publications: (*n*=10) meta-analyses and systematic reviews, (*n*=12) guidelines of care, (*n*=11) general evidence-based guidelines of wound care, (*n*=1) a framework for transitioning between moisture-retentive and NPWT.	Three algorithms were constructed based on NPWT[a] meta-analyses and systematic reviews, NPWT guidelines of care, general evidence-based guidelines of wound care and a framework for transitioning between moisture-retentive and NPWT.<br><br>12 participants participated in expert face validation of the algorithms. All had attended formal wound care classes, obtained wound care certification and practiced in acute, subacute, long-term or home care.
[42]	Data consisted of (*n*=24) questionnaires completed by (*n*=15) hospital nurses and (*n*=9) community nurses.	The clinical decision support model was constructed based on results from a questionnaire, which are not described further in the study.
[43]	Not reported in the study.	Literature review
5 [44]	Not reported in the study.	An extensive peer-review process in the form of a 3-phase Delphi[b] process (modified). Involved (1) An expert panel (wound experts n=11) from the United States, Canada and the United Kingdom developed the components of the clinical decision support model, (2) The clinical decision support model was disseminated to all ISTAP[c] members who distributed the statements to a wider group of reviewers (n=46), and (3) Written input from 46 reviewers and the ISTAP members generated the clinical decision support model.

[45]	Data consisted of (*N*=146) articles. (*n*=4) systematic reviews and (*n*=142) key publications.	A task force of clinical experts undertook (1) Review of the literature and identified evidence for support surface use in the prevention and treatment of pressure ulcers, (2) Developed supporting statements for essential components of the algorithm, (3) Developed a draft algorithm for support surface selection, and (4) Determined its face validity.
		A consensus panel (n=20) (1) Reviewed the draft algorithm and supporting statements, (2) Reached consensus on statements lacking robust supporting evidence, and (3) Modified the draft algorithm and evaluated its content validity.
