# Supplementary material for: Models Used in Clinical Decision Support Systems Supporting Healthcare Professionals Treating Chronic Wounds: Systematic Literature Review
Source: JMIR Diabetes. 2018 Jun 21;3(2):e11. doi: 10.2196/diabetes.8316 (PMC6238865; doi:10.2196/diabetes.8316)
Supplement: Multimedia Appendix 2 [file diabetes_v3i2e11_app2.pdf]

Table 7 An overview of the qualitative decision support models' accessibility of the inference engines, what type of wounds it focuses on and what type of professionals who have access.

**Reference No.**	**Accessibility of the inference engines of the system**	**Type of Wounds**	**Professionals, who have access to the system**
[40]	The clinical decision support model merges descriptive information provided by nurses using the National Pressure Ulcer Advisory Panel's criteria set up in a computer program as an algorithm in order to assign an accurate pressure ulcer stage. Nurses are required to select depth, color and characteristics of the pressure ulcer.<br>In the event that one or more characteristics are entered that do not meet the criteria for a particular pressure ulcer stage, the clinical decision support model indicates "no stage suggested". If no stage is suggested, the nurses can reselect criteria or manually select a pressure ulcer stage. The decision support model requires that the nurses take part in the process and are involved.	Pressure ulcers	Registered nurses and licensed practical nurses
[41]	Three different algorithms were presented. In the first algorithm, nurses are required to define the wound classification, define the goal of the wound care, define the goal of patient care plan, make a wound assessment, perform wound bed preparation /exudate and measure the wound depth. In the second and the third algorithms, nurses are required to perform wound bed preparation and examine the exudate, define the goal of patient care, measure the depth of the wound, perform wound bed preparation and examine the tissue, have anatomical considerations in mind and define the wound type and decide the goal of wound care. All three algorithms require that nurses take an active part in the processes and are involved.	Chronic wounds	All clinicians who undertake wound care for chronic wounds
[42]	The model used in the clinical decision support system requires nurses to take active part in the assessment of wounds.	Chronic wounds	Wound care links nurses who provide key support for the tissue viability service.
[43]	When surgeons have to choose between undertaking proactive (elective) surgery or reactive (nonelective) surgery, they can be helped by a clinical decision support model. The clinical decision support model helps to classify the surgery. The model requires that surgeons take an active part in the decisions.	Diabetic foot ulcers	Surgeons who undertake diabetic foot ulcer surgery
[44]	The model used in the clinical decision support system requires healthcare professionals to discover the skin tear, assess the bleeding, cleanse the skin tear, approximate and classify the wound edges, treat the cause, implement a prevention protocol, moisten wound healing, avoid trauma, protect peri wound skin, manage exudate, avoid infection and control pain.	Skin tears	All levels of staff and caregivers in multiple healthcare settings

[45]	Healthcare professionals can interact with three clinical decision support models (1) A skin and pressure ulcer risk assessment algorithm, (2) A prevention of pressure ulcers algorithm, and (3) A treatment of pressure ulcers algorithm.<br>Based on the risk of developing pressure ulcers or the presence of pressure ulcers, healthcare professionals follow pathways that guide clinical decision making for support surface use for pressure ulcer treatment.	Skin and pressure ulcers	Multiple healthcare professionals
